# A Combination of Astragaloside IV and Hydroxysafflor Yellow A Attenuates Cerebral Ischemia-Reperfusion Injury via NF-κB/NLRP3/Caspase-1/GSDMD Pathway

**DOI:** 10.3390/brainsci14080781

**Published:** 2024-07-31

**Authors:** Yongchun Hou, Zi Yan, Haitong Wan, Jiehong Yang, Zhishan Ding, Yu He

**Affiliations:** 1 Basic Medical School, Zhejiang Chinese Medical University, Hangzhou 310053, China; 2 Key Laboratory of TCM Encephalopathy of Zhejiang Province, Hangzhou 310053, China; 3 Department of Basic Medicine, Nanchang Medical College, Nanchang 360000, China

**Keywords:** astragaloside IV, hydroxysafflor yellow A, cerebral ischemia-reperfusion injury, NF-κB/NLRP3/Caspase-1/GSDMD pathway

## Abstract

Cerebral ischemia-reperfusion injury (IRI), occurring after blood supply restoration, contributes significantly to stroke-related deaths. This study explored the combined impact and mechanisms of astragaloside IV (AS-IV), hydroxysafflor yellow A (HSYA), and their combination in mitigating IRI. Male Sprague–Dawley (SD) rats were randomized to the Sham, MCAO, MCAO+AS-IV, MCAO+HSYA, and MCAO+AS-IV+HSYA groups. Neurological deficits and cerebral infarction were examined after restoring the blood supply to the brain. Pathomorphological changes in the cerebral cortex were observed via HE staining. IL-1β and IL-18 were quantified using ELISA. The expression of NF-κB and GSDMD in the ischemic cerebrum was analyzed using immunohistochemistry. The expression levels of NLRP3, ASC, IL-1β, Caspase-1, and GSDMD in the ischemic cerebrum were evaluated using Western blot. The MCAO+AS-IV, MCAO+HSYA, and MCAO+AS-IV+HSYA groups exhibited notably better neurological function and cerebral infarction compared with the MCAO group. The combined treatment demonstrated superior brain tissue injury alleviation. Reductions in NF-κB, GSDMD positive cells, and NLRP3/ASC/IL-1β/Caspase-1/GSDMD protein expression in the ischemic brain were significantly more pronounced with the combined therapy, indicating a synergistic effect in countering cerebral IRI via the NF-κB/NLRP3/Caspase-1/GSDMD pathway inhibition of cell pyroptosis-induced injury.

## 1. Introduction

Ischemic stroke occurs when there is a vascular obstruction in a brain artery, causing cerebral ischemic hypoxia and brain dysfunction [1]. Ischemic stroke has a complex pathogenesis involving the production of free radical oxygen species (ROS), calcium overload, dysfunctional energy metabolism, cell apoptosis, and inflammation, leading to permanent central nervous system (CNS) damage and long-term disability [2]. The timely restoration of the blood supply in the ischemic area is critical to reduce the patient’s risk of death, but blood supply restoration also causes injury, leading to cerebral ischemia-reperfusion injury; the CNS needs to be protected from further injury after reperfusion [3]. Although many studies have investigated potential therapeutic strategies to alleviate ischemia-reperfusion injury, targeted drugs are presently lacking due to a poor understanding of the mechanisms underlying ischemia-reperfusion injury. Developing targeted drugs with known mechanisms of action may reduce ischemia-reperfusion injury.

The NF-κB/NLRP3/Caspase-1/GSDMD pathway is involved in cerebral ischemia-reperfusion injury, and its related molecules have become experimental targets for alleviating cerebral ischemia-reperfusion injury [4]. The Nod-like receptor (NLR) family is a multiprotein complex capable of recognizing signals such as pathogen-associated molecular patterns and risk factor-related model molecules. It is composed of NLR protein 3 (NLRP3), apoptosis-associated speck-like protein (ASC), and Caspase-1. Caspase-1 is an important part of the innate immune system, and activated Caspase-1 can cleave the Gasdermin D protein (GSDMD) and release the inflammatory cytokines interleukin-1 β (IL-1β) and IL-18, thereby inducing cell pyroptosis [5]. NF-κB is a transcription factor that controls the formation of the NLRP3 inflammasome. Hence, the NF-κB/NLRP3 inflammasome signaling pathway is a key signaling pathway associated with ischemic stroke [6]. Several chemical compounds targeting the NLPR3 pathway have been investigated in many experimental studies, with promising outcomes [7]. Unfortunately, most of these compounds have been restricted to preclinical phases without entering proof-of-concept and large human clinical trials. Various traditional Chinese medicine (TCM) drugs have also been investigated in many experimental trials to alleviate ischemia-reperfusion injury [8,9,10]. TCM drugs are effective and safe due to their multiple functions and minimal side effects compared with chemical drugs. 

Astragalus and safflower have been used in experimental studies on the treatment of ischemic stroke [11,12,13,14]. Astragalus has the property of tonifying qi, and safflower promotes blood circulation. The compatibility of these two herbs facilitates their use in combination with TCM for treating various disorders [15]. Astragaloside IV (AS-IV) and hydroxysafflor yellow A (HSYA) are the main active components of Astragalus and safflower [16], respectively. In previous studies, AS-IV alleviated oxidative stress injury, inhibited cell apoptosis, promoted vascular remodeling and regeneration, and showed a protective effect on cerebral ischemia-reperfusion injury [17,18,19,20]. HSYA was found to increase cerebral blood flow, reduce infarct size, regulate related signal pathways, antagonize endoplasmic reticulum stress and oxidative stress, and reduce inflammatory cascade after cerebral ischemia [14,21,22]. However, there is little data about the combination of AS-IV and HSYA in treating cerebral ischemia-reperfusion injury [20].

This study aimed to examine the effects of the combination of AS-IV+HSYA compared with AS-IV and HSYA on ischemia-reperfusion injury, using a rat model of focal cerebral ischemia-reperfusion injury induced by MCAO. This study planned to mainly evaluate the effects of AS-IV and HSYA on NLRP3 inflammasomes after ischemic stroke and further elucidate the anti-pyroptotic effects via the NLRP3/Caspase-1/GSDMD pathway.

## 2. Materials and Methods

### 2.1. Chemicals and Reagents

AS-IV (molecular formula: C_41_H_68_O_14_; molecular weight: 784.97; purity: ≥98%; batch number: kr202107025as) and HSYA (molecular formula: C_27_H_32_O_16_; molecular weight: 612.54; purity: ≥98%; batch number: kr202102003qa) were purchased from Kerui Instrument Distribution Department, Baoji Shaanxi Province, China; 2,3,5-triphenyltetrazolium chloride (TTC) and diaminobenzidine were provided by Nanjing Jian Cheng Bioengineering Institute (Jian Cheng, Nanjing, China); antibody diluent was purchased from Bitian Biotechnology Co., Ltd. (Bitian, Shanghai, China); interleukin-1β (IL-1β) and interleukin-18 (IL-18) detection kits were from Beijing Zhongshan Jin Qiao Co., Ltd. (Beijing, China); hematoxylin eosin (HE) staining solution was from Shanghai Sanaisi Reagent Co., Ltd. (Shanghai, China) (batch No. 20200312); β-actin was purchased from the USA (GIBCO, NewYork, NY, USA) (batch No. 66056-1-ig); NLRP3, Caspase-1, IL-1β, ASC, and GSDMD primary antibodies were purchased from Abcam, Cambridge, UK; Secondary antibodies were purchased from GIBCO (NewYork, NY, USA); malondialdehyde (MDA) and superoxide dismutase (SOD) kits were purchased from Nan Jing Jiancheng Institute of Bioengineering (Nanjing, China).

### 2.2. Experiment Animals

Specific pathogen-free (SPF) healthy SD male adult rats weighing 280 ± 20 g were obtained from the animal experiment center of Zhejiang Chinese Medical University. The study protocol was approved by the Animal Experiment Review Committee of Zhejiang Chinese Medical University (approval number: syxk 2019-0043). The experiments were monitored by the Animal Experiment Review Committee of Zhejiang Chinese Medical University. All procedures complied with the National Institutes of Health guidelines for the care and use of laboratory animals. 

Before the onset of the experiment, the rats were acclimatized to a temperature of 20–25 °C, ambient humidity of 40–45%, and 12/12-h light/dark cycles. The rats had free access to food and water and adaptive feeding for 3 days.

### 2.3. Establishment of Ischemia-Reperfusion Injury Model by MCAO and Animal Grouping

The rats were fasted (food and water) 12 h before surgery. After weighing, the animals were anesthetized with Zoletil50 (50 mg/kg, Virbac, Carros, France). The cerebral ischemia-reperfusion injury model by middle cerebral artery occlusion (MCAO) was performed using the suture method [23]. The rats were placed supine on a warm pad (maintaining the core temperature at about 37.0 °C). An incision was made in the middle of the neck. The sternocleidomastoid and sternotoglossus muscles were bluntly separated, the common carotid artery (CCA) was fully exposed, and the external carotid artery (ECA) and internal carotid artery (ICA) were separated. The proximal end of the CCA and the root of the ECA were ligated with a silk thread, the ICA was clamped with micro artery clamp, and a “V” shaped cut was made at the proximal bifurcation of the CCA. The smooth round head end of the threaded plug was gently pushed from the incision while the internal carotid artery clamp was opened, and the threaded plug entered the starting end of the middle cerebral artery along the internal carotid artery to completely block the blood supply to the brain. The threaded plug was fixed and kept stable. After 1 h, the threaded plug was gently pulled out to achieve reperfusion. The incision was closed, and the access point was sutured. In the Sham group, a similar surgical procedure was performed, except for threaded plug insertion. After anesthesia recovery, the rats showed the Mer sign in the right eye and left forelimb adduction flexion during tail lifting, turning, or dumping to the left during walking (except for the Sham group). In addition, no skull base bleeding was observed, indicating that the model was successfully developed.

**Figure 1:**
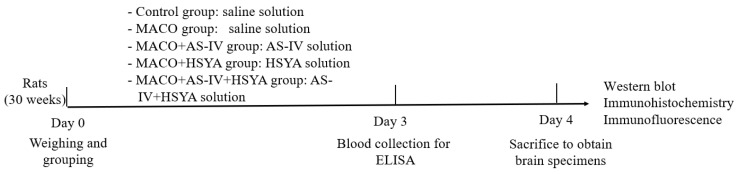
Study flowchart.

The study design is presented in Figure 1. The rats were randomly divided into the Sham, MCAO, MCAO+AS-IV, MCAO+HSYA, and MCAO+AS-IV+HSYA groups, with six rats in each group. According to previous studies [8,9,24], the MCAO+AS-IV group was injected with 40 mg/kg AS-IV + 1 mL of 0.9% saline, and the MCAO+HSYA group was injected with 20 mg/kg HSYA + 1 mL of 0.9% saline. The MCAO+AS-IV+HSYA group was administered 40 mg/kg AS-IV + 20 mg/kg HSYA. All injections were administered into the caudal vein once daily for 3 consecutive days. The Sham group was administered a similar volume of sterile 0.9% saline. All animals had free access to food and water and were kept in the appropriate environment. The rats were anesthetized with a mixture of ketamine and pentobarbital when blood was collected.

### 2.4. Evaluation of Neurological Deficits

The neurological deficits of rats were evaluated on a 5-point scale [25]: 4 points indicate that the rats are unable to walk independently or are in a coma; 3 points indicate that the rats reverse left; 2 points indicate that the rats turn left; 1 point indicates that the left claw cannot be fully extended; and 0 indicates that there is no neurological dysfunction. 

### 2.5. Analysis of Infarct Volume in the Brain

The brain specimens were cut into five coronal sections of 2 mm, and then stained in 2% TTC at 37 °C for 30 min. The TTC-stained sections were imaged and quantified using Image Pro-Plus version 6.0 in a blinded fashion. The brain infarct volumes were determined as the ratio to the contralateral hemisphere volume. The percentage of infarct volume was calculated by the following equation: [(total contralateral hemispheric volume) − (total ipsilateral hemispheric stained volume)]/(total contralateral hemispheric volume) × 100%.

### 2.6. Histopathological Examination

After anesthesia, the rats were perfused with normal saline (4 °C). The brain specimens were collected and fixed in 4% paraformaldehyde (PFA) for 24 h. Paraffin sections of 3–4 µm thickness were cut and stained with hematoxylin-eosin (HE) and Nissl staining for histopathological examination. The ischemic coronal plane of each rat was viewed using a digital CCD camera connected to a fluorescence microscope computer imaging system (MCID). Image J software (version 1.8.0.345) was used to quantify the fluorescence intensity.

### 2.7. Water Content of Brain Tissue

The rats were anesthetized and sacrificed by decapitation to harvest the brain. The cerebral cortex was collected and drained with filter paper to remove surface blood and cerebrospinal fluid. The wet weight of the brain was measured with an analytical balance. Then, the brain was baked in an oven at 100 *°C* for 24 h to obtain its dry weight. The degree of cerebral edema was calculated using the formula brain water content % = (wet weight − dry weight)/wet weight × 100%.

### 2.8. Immunohistochemical Staining of Brain Tissue

Paraffin-embedded brain sections were used for immunohistochemistry. Brain sections with a thickness of 3–4 μm were incubated with the primary antibody (PE-labeled α-tubulin mouse monoclonal antibody; 1:2500) at 37 °C for 1 h. The sections were incubated with the secondary antibody (goat anti-rabbit IgG antibody; 1:5000) at 37 °C for 30 min. Diaminobenzidine (DAB) was used as the color-developing agent to produce a color reaction. The sections were counterstained with hematoxylin and dehydrated with alcohol and xylene. Images of the ischemic cortex were captured using an optical microscope. The positive staining was shown in brown. Caspase-1 positive cells were quantified using Image-Pro Plus software (version 6.0) to determine the integrated density (IOD).

The brain tissue sections were also stained with fluorescein-conjugated antibodies against NF-κB and NLRL3, counterstained with DAPI, and sealed with an antifade mounting medium. The stained ischemic cortex was viewed under a fluorescence microscope (Eclipse C1, Nikon, Tokyo, Japan). Image J software (version 1.8.0.345) used to quantify the fluorescence intensity.

### 2.9. Assessment by ELISA

Blood was collected by cardiac puncture in clot-activated tubes, allowed to stand for 10 min, and centrifuged to obtain serum. The serum levels of MDA, SOD, IL-1β, and IL-18 were measured using commercial ELISA kits.

The brain homogenate was allowed to stand for 10 min, and the supernatant was collected. The contents of MDA and SOD antioxidant enzymes and the levels of TLR4, IL-1β, and IL-18 in the cerebral ischemic cortex of MCAO rats were detected by ELISA.

### 2.10. Western Blot Analysis

Total proteins were extracted from brain samples using the RIPA buffer and quantified using the BCA method. Then, equal amounts of proteins were mixed with the same volume of protein loading buffer, separated by SDS-PAGE, and transferred to PVDF membranes. The membranes were washed with TBST and blocked with 3% skimmed milk for 3 h. The primary antibodies against NLRP3, ASC, Caspase-1, and GSDMD (all diluted 1: 1000) were added and incubated at 4 °C overnight. β-actin (diluted 1: 10,000) was used for normalization. After washing the membranes, peroxidase-labeled goat anti-rat IgG (1: 3000) and goat anti-rabbit IgG (1: 10,000) were added, incubated for 1 h, and the color was developed by electrochemiluminescence (ECL). The relative expression levels of the target proteins were calculated based on the ratio of the gray value of the target protein band to the gray value of the β-actin band.

### 2.11. Statistical Analysis

SPSS 24.0 statistical software was used for statistical analysis. Measurement data were expressed as mean ± standard deviation. A one-way analysis of variance was performed to compare variables between multiple groups, and the Student–Newman–Keuls-q (SNK-q) test was used to compare pairs between groups. A *p*-value of <0.05 was considered statistically significant.

## 3. Results

### 3.1. AS-IV+HSYA Improves Neurological Deficits and Reduces Cerebral Infarction after Cerebral Ischemia-Reperfusion

**Figure 2:**
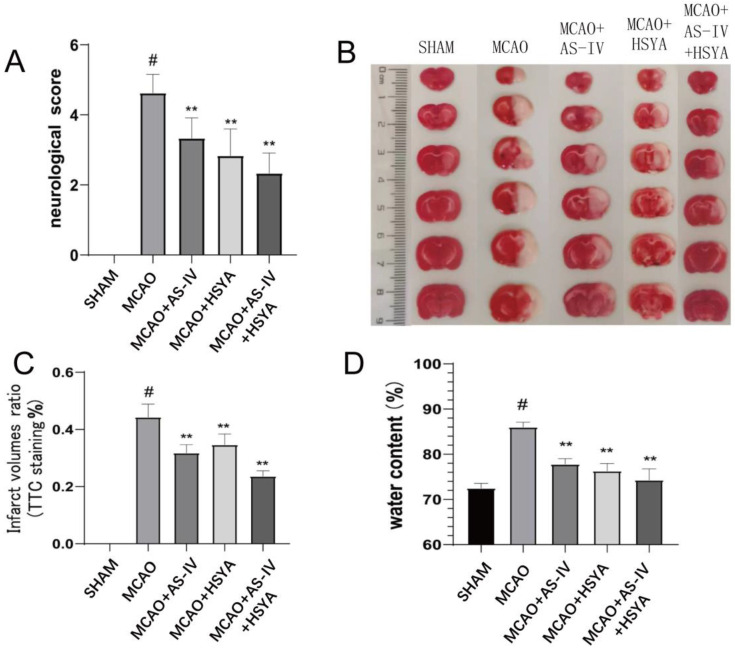
Effects of AS-IV, HSYA, and their combination on nerve function, cerebral infarction, and cerebral edema. (**A**) Neurological function deficit scores on the third day after reperfusion. Data are presented as mean ± standard deviation (*n* = 6). (**B**) TTC staining of representative cerebral infarction on the coronal plane of the brain. (**C**) The infarct ratios were calculated from the TTC staining images. Data are presented as mean ± standard deviation (*n* = 6). (**D**) The brain water content represents cerebral edema. Data are presented as mean ± standard deviation (*n* = 6). ** compared with the MCAO group (*p* < 0.01), # compared with the Sham group (*p* < 0.01).

Neurological deficits and cerebral infarction were examined to determine the alleviation of ischemia-reperfusion injury by AS-IV+HSYA. As shown in Figure 2 and Table 1, the rats in the MCAO group had significant neurological damage 3 days after reperfusion (*p* < 0.01). The AS-IV, HSYA, and AS-IV+HSYA treatments were effective in alleviating nervous system injury compared with the MCAO group (*p* < 0.05) (Figure 2A). Compared with the Sham group, the total infarct volume in the MCAO group was significantly larger (*p* < 0.01). The infarct volume was significantly decreased in the drug intervention groups (*p* < 0.01) (Figure 2B,C). Compared with the Sham group, cerebral edema volume in the MCAO group was significantly greater (*p* < 0.01). In the MCAO+AS-IV, MCAO+HSYA, and MCAO+AS-IV+HSYA groups, cerebellar edema was effectively reduced compared with the MCAO group (*p* < 0.01) (Figure 2D).

### 3.2. AS-IV+HSYA Alleviates Brain Tissue and Cell Damage in Rats after Cerebral Ischemia-Reperfusion Injury

**Figure 3:**
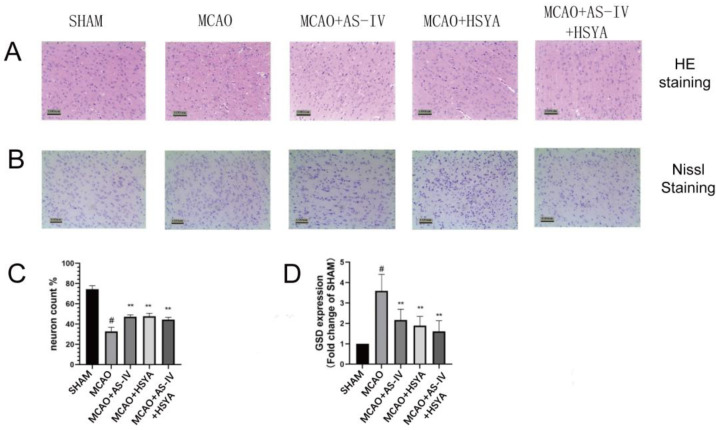
The effects of AS-IV, HSYA, and their combination on histopathological changes, cell survival, and death in the ischemic cerebral cortex. (**A**) Representative HE-stained images of the ischemic cerebral cortex of rats from each group. Bar = 100 µm. (**B**) GSD positive cells by Nissl staining in the ischemic cerebral cortex after reperfusion. Bar = 100 µm. (**C**,**D**). The quantitative evaluation of intact neurons and GSD-positive cells. Data are presented as mean ± standard deviation (*n* = 6), ** compared with the MCAO group (*p* < 0.01), # compared with the Sham group (*p* < 0.01).

The brain sections were examined to determine the effect of AS-IV plus HSYA in alleviating neuronal cell and tissue damage after cerebral ischemia-reperfusion injury. HE and Nissl staining revealed obvious neuronal damage in the MCAO group (Figure 3A,B). The rats in the MCAO+AS-IV group, MCAO+HSYA group, and MCAO+AS-IV+HSYA group also displayed similar pathological changes to those of the MCAO group, but the histopathological changes were less severe. In the MCAO+AS-IV+HSYA group, histopathological damage was obviously reduced. Compared with the Sham group, the MCAO group had significantly fewer intact neurons (*p* < 0.01). The numbers of intact neurons in the MCAO+AS-IV, MCAO+HSYA, and MCAO+AS-IV+HSYA groups were significantly higher compared with the MCAO group (*p* < 0.01) (Figure 3C and Table 2). Compared with the Sham group, the expression of GSD in the MCAO group was significantly increased (*p* < 0.01) and significantly decreased in the treatment groups (*p* < 0.01) (Figure 3D and Table 2).

### 3.3. The Reduction in Inflammatory Factors in the Cerebral Cortex and Blood by AS-IV+HSYA after Cerebral Ischemia-Reperfusion Injury

**Figure 4:**
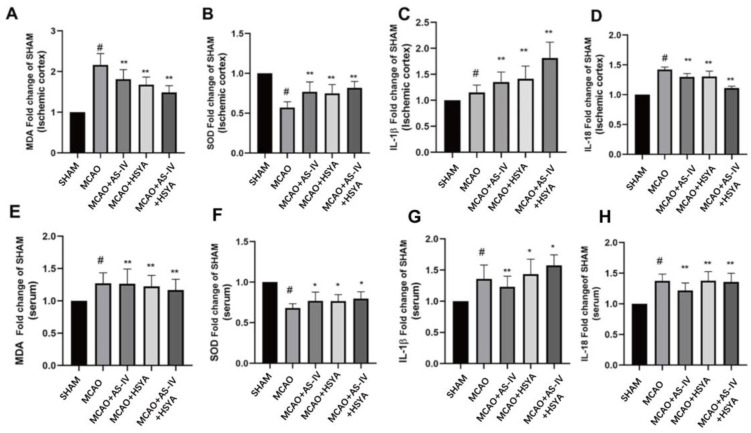
The effects of AS-IV, HSYA, and their combination on oxidative stress factors from the ischemic cortex and serum. (**A**–**H**) The contents of MDA, SOD, IL-1β, and IL-18 in the ischemic cortex and serum of rats from each experimental group. Data are presented as mean ± standard deviation (*n* = 6), * compared with the MCAO group (*p* < 0.05), ** compared with the MCAO group (*p* < 0.01), # compared with the SHAM group (*p* < 0.01).

Inflammatory factors were evaluated in the cerebral cortex and serum using ELISA to ascertain the alleviation of cerebral ischemia-reperfusion injury by AS-IV+HSYA. As shown in Figure 4, Table 3, and Table 4, compared with the Sham group, the MDA and SOD levels in the ischemic cortex in the MCAO group were significantly changed (*p* < 0.05). The AS-IV treatment, HSYA treatment, and combined treatment of both could bring MDA and SOD levels in the ischemic cortex back to Sham level, and all treatment groups had significant effects (*p* < 0.01). Similarly, the serum MDA levels increased significantly in the MCAO group compared with the Sham group (*p* < 0.05) and decreased in all treatment groups compared with the MCAO group (*p* < 0.01). The serum SOD levels decreased in the MCAO group compared with the Sham group (*p* < 0.05) and increased in all treatment groups compared with the MCAO group (*p* < 0.05). We also detected the levels of IL-1β and IL-18 in all groups, and all the treatment groups showed significant differences (*p* < 0.05 or *p* < 0.01), suggesting that AS-IV treatment, HSYA treatment, and AS-IV+HSYA treatment may play a role by influencing the levels of inflammatory factors.

### 3.4. The Effects of AS-IV+HSYA on NLRP3/Caspase-1/GSDMD Signaling and Inflammasome-Related Molecules

**Figure 5:**
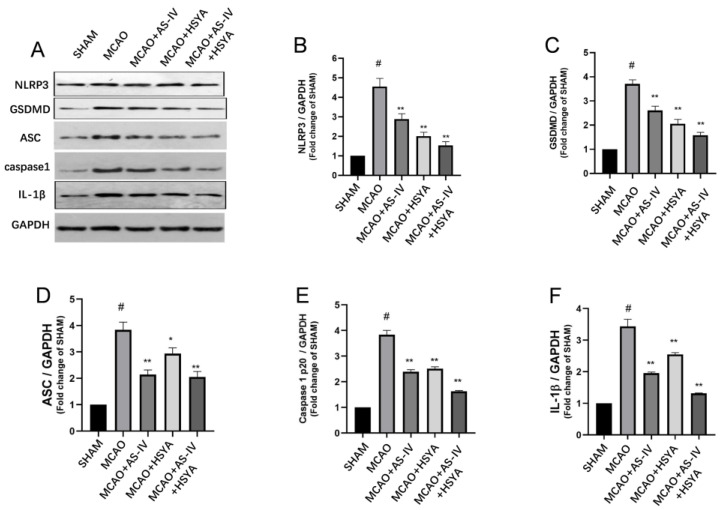
The effects of AS-IV, HSYA, and their combination on NLRP3/Caspase-1/GSDMD pathway and inflammasome-related proteins. (**A**–**F**) WB representative images and semi-quantifications of NLRP3, GSDMD, ASC, and Caspase-1. Data are expressed as mean ± standard deviation (*n* = 3). * Compared with the MCAO group (*p* < 0.05), ** compared with the MCAO group (*p* < 0.01), # compared with the Sham group (*p* < 0.01).

**Figure 6:**
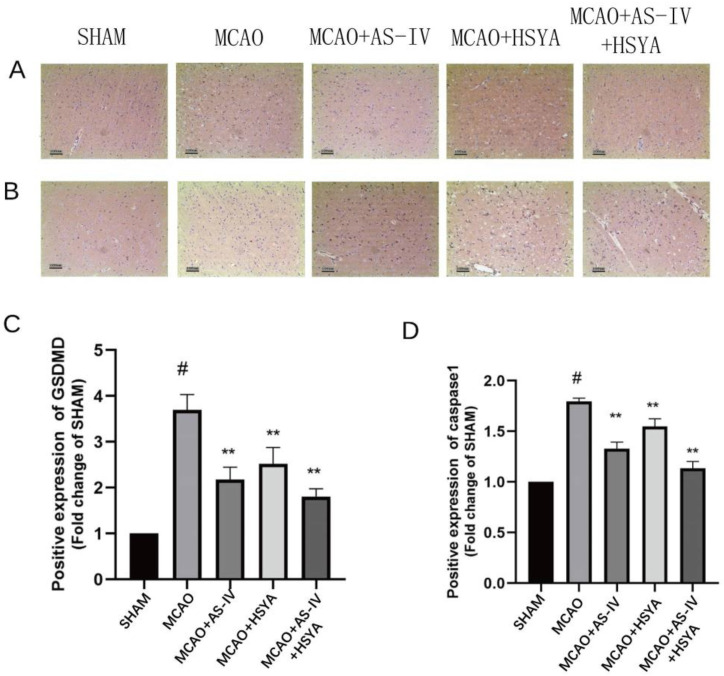
The effects of AS-IV, HSYA, and their combination on NLRP3/Caspase-1/GSDMD pathway and inflammasome-related proteins. (**A**,**B**) Immunohistochemical staining images of GSDMD and Caspase-1, bar = 100 µm. (**C**,**D**) Quantitative analysis of GSDMD and Caspase-1 positive expression. Data are presented as mean ± standard deviation (*n* = 3), ** compared with the MCAO group (*p* < 0.01), # compared with the Sham group (*p* < 0.01).

In order to elucidate the effect of AS-IV plus HSYA on the NLRP3/Caspase-1/GSDMD signaling pathway, associated proteins, GSDMD (cleaved), ASC, caspase-1p20, and IL-1β were analyzed using Western blot. Compared with the Sham group, the GSDMD (cleaved) level in the MCAO group was increased, and the GSDMD (cleaved) level in the MCAO+AS-IV, MCAO+HSYA, and MCAO+AS-IV+HSYA groups was significantly reduced compared with the MCAO group (*p* < 0.01, Figure 5C). The expression trends of ASC, caspase-1p20, and IL-1β were similar to that of GSDMD (cleaved) (*p* < 0.01) (Figure 5 and Supplementary Tables S1 and S2). The GSDMD protein was mainly localized in the nuclei of apoptotic cells/apoptotic-like bodies in the brain tissue (Figure 6 and Supplementary Table S3). Caspase-1 was mainly localized in the brain endothelium, neurons, and glial cells, and the activated Caspase-1 was brownish yellow, seen from cytoplasm to nucleus. No significant Caspase-1-positive cells were found in the brain tissue of the Sham-operated rats. Compared with the Sham group, the expression of Caspase-1 in the brain tissue from the MCAO group was significantly increased (*p* < 0.01), and the expression of Caspase-1 in the brain tissue from the other groups was significantly decreased (*p* < 0.01).

### 3.5. The Effect of AS-IV+HSYA on the Contents of Inflammation-Related Protein Molecules

**Figure 7:**
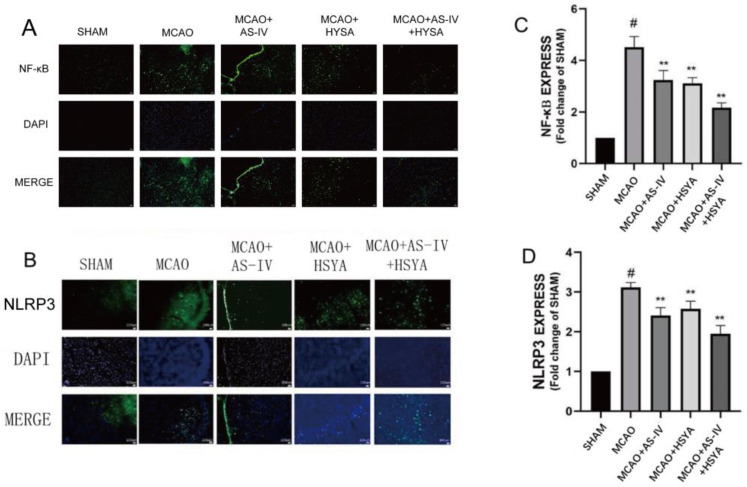
The effects of AS-IV, HSYA, and their combination on the extent of expression of the inflammasome-related proteins. (**A**–**D**) Immunofluorescence images and quantitative analysis of NF-κB and NLRP3 expression. Data are presented as mean ± standard deviation (*n* = 3), bar = 100 µm. ** compared with the MCAO group (*p* < 0.01), # compared with the Sham group (*p* < 0.01).

Inflammatory cytokines and mediators play a key role in inflammatory responses. Since the activation of NLRP3 inflammasomes is an upstream event, changes in NF-kB protein-like proteins in the cytosol directly affect the activation of NLRP3 inflammasomes and cause neuronal damage, releasing IL-1 β, IL-18, and other protein factors. As shown in Figure 6 and Table 5, compared with the Sham group, the levels of NF-kB and NLRP3 proteins in the MCAO group were significantly increased (*p* < 0.01). With the drug intervention, the levels of NF-kB and NLRP3 proteins in the MCAO+AS-IV and MCAO+HSYA groups were significantly decreased (*p* < 0.05). The MCAO+AS-IV+HSYA and MCAO groups had a significant decrease in the levels of these proteins (*p* < 0.01). Combined treatment of AS-IV and HSYA reduced or reversed the expression of proteins of the NF-κB/NLRP3/Caspase-1/GSDMD pathway in the cerebral cortex of the MCAO model rats (Figure 7). 

## 4. Discussion

Ischemic stroke is very common in clinical practice, accounting for about 87% of all strokes, causing lasting brain damage, long-term disability, or even death [12,26,27]. The current main therapeutic strategy remains the rapid restoration of blood supply by recombinant tissue plasminogen activator (rt-PA) or mechanical removal of the clot. Nevertheless, the therapeutic window is narrow, and there is a requirement for specialized equipment and experience. However, this treatment has time and space limitations. Therefore, the search for other effective treatment options is very necessary. 

In TCM, herbal drugs are effectively combined to treat various disorders [28,29]. When two herbal medicines are combined, the active ingredients should be compatible and synergistic in treating ailments. In TCM, the effective combination of herbal drugs has been achieved from the long-term clinical medication experience of past-generation physicians [30,31]. 

The MCAO model is widely used in experimental research and closely simulates cerebral ischemia-reperfusion. Animals with cerebral ischemia and reperfusion show movement disorders and neuropathological changes [29,32]. In this study, neurological deficit and cerebral infarction were more important in the MCAO model rats than in Sham rats, demonstrating that cerebral ischemic stroke was successfully developed in rats. The AS-IV, HSYA, and AS-IV+HSYA treatments improved the neurological deficit and cerebral infarction area after MCAO. It is suggested that these treatments, either alone or in combination, could significantly reduce cerebral ischemia-reperfusion injury. The present study showed that AS-IV and HSYA could alleviate brain tissue and cell damage after ischemia-reperfusion injury, consistent with the literature [33,34,35]. Still, the exact mechanisms remain to be determined [36]. 

Indeed, the pathogenesis of cerebral ischemia-reperfusion injury is complex [37,38,39]. The pathophysiological mechanisms of cerebral ischemia-reperfusion injury include oxidative stress, leukocyte infiltration, mitochondrial breakdown, platelet activation and aggregation, complement activation, and blood–brain barrier disruption, in which the inflammatory response is inherent [33,40,41]. Oxidative stress occurs due to the imbalance between oxidants and antioxidants and excessive production of reactive oxygen species (ROS). Excessive production of ROS causes neuronal necrosis, releasing many inflammatory factors such as IL-1β and IL-18. Secondary injury in stroke also involves elevated proinflammatory cytokines and neuroinflammation [42]. Increased levels of MDA, SOD, IL-1β, and IL-18 can indirectly reflect the degree of damage. This study determined the above-mentioned indicators in the ischemic cortex and serum of rats. Compared with the Sham group, the oxidative stress and inflammation indicators were significantly changed in the ischemic cortex and serum in the MCAO group. The drug intervention caused significant changes in these indexes in the ischemic cortex and serum in each treatment group, compared with the MCAO group. Furthermore, the changes in the MCAO+AS-IV+HSYA group were more obvious except for IL-18, suggesting that drugs may have increased the permeability of the blood–brain barrier and reduced the damage. Those anti-inflammatory changes from the study drugs are supported by the literature [35,43]. 

Pyroptosis is an inflammatory form of programmed cell death. The canonical pyroptosis pathway depends on activated Caspase-1, which hydrolyzes the membrane protein GSDMD at the n-terminal and c-terminal ends. The N-terminus of GSDMD oligomerizes to form pores on cell membranes [24,32,44]. Cell edema and pyroptosis destroy the integrity of the cell membrane, releasing cell contents [29,45,46]. Among the known inflammasomes, the NLRP3 inflammasome is the most widely studied [47]. The inflammatory effect caused by the activation of the NLRP3 inflammasome plays an important role in cerebral ischemia-reperfusion injury [48,49]. NF-κB, as an upstream activator of the NLRP3 inflammasome, controls the transcription of inflammation-related genes [50,51,52]. Aggregated and activated inflammasomes include three domains, NLRP3, ASC, and Caspase-1, and cause increased levels of IL-1β and IL-18. Additionally, GSDMD, as an important factor of pyroptotic death execution downstream of Caspase-1 (canonical inflammasome), directly participates in brain ischemia-reperfusion injury. 

In the Western blot and immunofluorescence analyses, the expression levels of pyroptosis-related factors, such as NF-κB, NLRP3, GSDMD, ASC, Caspase-1, and IL-1β, were increased significantly after cerebral ischemia-reperfusion injury [45,53,54]. Combined treatment of AS-IV and HSYA reduced or reversed the expressions of proteins of the NF-κB/NLRP3/Caspase-1/GSDMD pathway in the cerebral cortex of the MCAO model rats. It has been previously shown that AS-IV can inhibit the NLRP3 inflammasome [55]. HSYA can inhibit the NLRP3 inflammasome by binding to xanthine oxidase [43,56]. Considering the involvement of NLRP3 in cerebral ischemia-reperfusion damage, the effect of AS-IV could explain why AS-IV+HSYA was effective. Cerebral ischemia-reperfusion damage was alleviated to different degrees, suggesting that the NF-κB/NLRP3/Caspase-1/GSDMD signaling pathway is involved in the pathogenesis and healing of ischemic stroke. The AS-IV+HSYA treatment may have protected the rat cortical cells from pyroptosis through the NF-κB/NLRP3/Caspase-1/GSDMD pathway. Furthermore, AS-IV+HSYA significantly regulated the expression levels of NF-κB, NLRP3, GSDMD, ASC, Caspase-1, and IL-1β more than the AS-IV or HSYA, although they did not differ statistically. The present study only skimmed the surface of the potential mechanisms of AS-IV+HSYA for the management of stroke. Several other pathways and mechanisms could be involved in their effects on cerebral ischemia-reperfusion damage.

**Figure 8:**
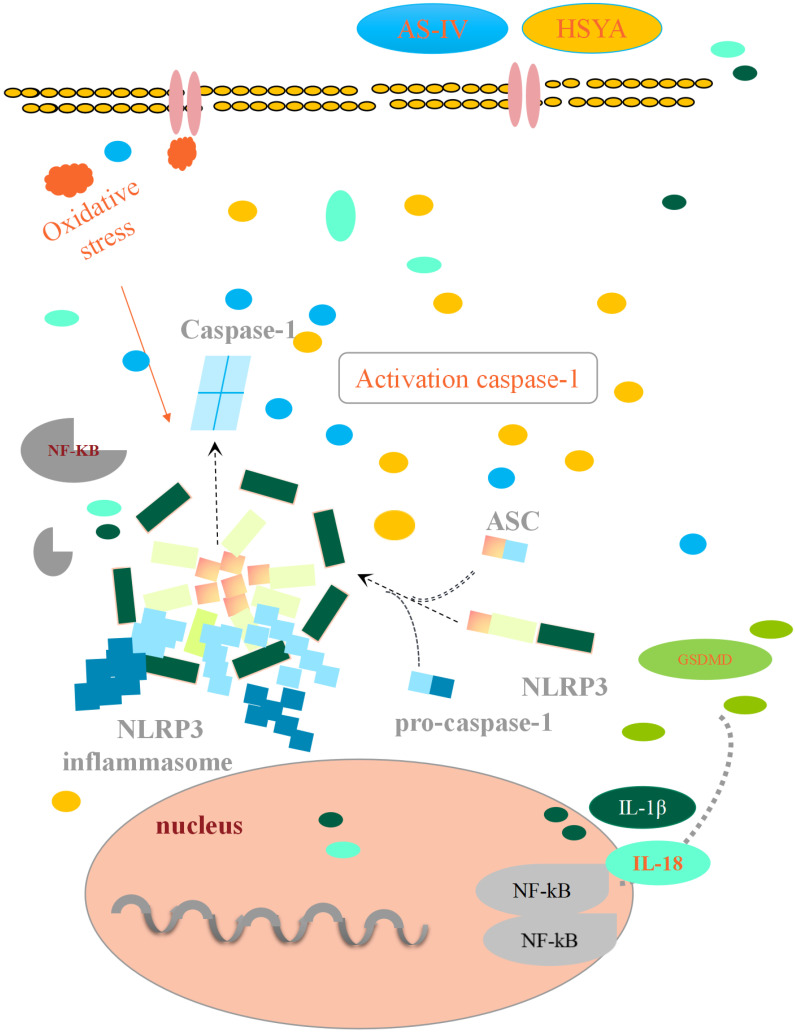
The diagrammatic representation of the protective effect of AS-IV and HSYA on cerebral ischemia-reperfusion injury. The cerebral ischemia-reperfusion triggers focal cellular death by activating the GSDMD-Caspase-1 signaling pathway, leading to the death of the brain neurons. The cerebral edema caused by the injury aggravates the microvascular perfusion insufficiency in the damaged cerebral cortex, further aggravating the injury. AS-IV, HSYA, and their combination can reverse or reduce brain damage to varying levels; combined formulation shows a more pronounced curative effect.

Therefore, the NF-κB/NLRP3/Caspase-1/GSDMD pathway is involved in cerebral ischemia-reperfusion damage after stroke. The AS-IV+HSYA combination may alleviate cerebral ischemia-reperfusion injury in rats by downregulating the activation of NF-κB, which would, in turn, decrease GSDMD, NLRP3, and Caspase-1 activation and prevent pyroptosis (Figure 8). Still, how AS-IV and HSYA can decrease NF-κB requires additional study.

This study had some limitations. Firstly, the TCM compounds were administered only for 3 days, and the effect of longer treatments (e.g., 7 and 14 days) was not explored. Secondly, cerebral ischemia-reperfusion not only causes inflammation and oxidative stress but also cell apoptosis, mitochondrial dysfunction, and other ill effects, which were not investigated in this study. Thirdly, the dosage of the two drugs in the MCAO+AS-IV+HSYA group mainly came from many previous single-drug experiments, which might not have accurately optimized the dosage of the two drugs when combined. Further research is necessary to evaluate the optimal doses of AS-IV and HSYA to alleviate cerebral ischemia-reperfusion injury. Fourthly, the levels of MDA, SOD, IL-1β, and IL-18 were not analyzed in whole brain samples across all groups. Fifthly, the degree of cerebral ischemic injury may not have been consistent when the animal model was made, and there may have been extreme cases of great difference between groups; this may have had an impact on the results of the experiment. Finally, the present study was not designed to determine the exact mechanisms involved in the effects of AS-IV and HSYA in stroke, but only to explore a few possibilities. Additional in vitro and in vivo studies are necessary. AS-IV and HSYA appear to have additive or synergistic effects in some experiments but these did not reach statistical significance. It is possible that the small sample size could explain that.

AS-IV, HSYA, and AS-IV+HSYA reduced the infarcted area of the cerebral cortex and cerebral edema and increased the permeability of the blood–brain barrier. This combination may alleviate cerebral ischemia-reperfusion injury in rats by downregulating the signaling molecules of the NF-κB/NLRP3/Caspase-1/GSDMD pathway. Furthermore, the effect of combined medication was better in lessening the injury than a single herbal compound. In conclusion, the combined formulation of AS-IV and HSYA was found to be effective in treating rats with cerebral ischemia-reperfusion injury.

## 5. Conclusions

In conclusion, our study showed that astragaloside IV, hydroxysafflor yellow, and the combination of astragaloside IV and hydroxysafflor yellow A could reduce cerebral ischemic volume and cerebral edema area in rats and improve cerebral ischemia-reperfusion injury. The effect of combined therapy was better than that of single therapy. The mechanism may be downregulation of NFΚB/NLRP3/Caspase-1/GSDMD pathway protein expression and regulation of NLRP3 inflammasome-mediated pyroptosis associated with cerebral ischemia-reperfusion. Therefore, the combination of astragaloside IV and hydroxysafflor yellow A is a new effective candidate that has great potential in I/R therapy.

## Figures and Tables

**Table 1:** Effects of each experimental group on neurological deficits, TTC infarct, and water content in brain tissue (x ± s, *n* = 6).

Group	Neurobehavioral Score				TTC Infarct %				Water Content/%			
	Mean	Sd	*t* Test	Sign	Mean	Sd	*t* Test	Sign	Mean	Sd	*t* Test	Sign
Sham	0	0	0	^#^	0	0	0	^#^	42.17	1.47	0	^#^
MCAO	2.83	0.52	-		0.44	0.05	-		83.67	2.34	-	
MCAO+AS-IV	1.75	0.61	0.002	**	0.32	0.03	0	**	70.17	5.12	0	**
MCAO+HSYA	1.58	0.49	0	**	0.35	0.04	0	**	68.17	6.82	0	**
MCAO+AS-IV +HSYA	1.42	0.49	0	**	0.24	0.02	0	**	57.33	2.58	0	**
F	27.35				180.81				82.97			
*p*	0				0				0			

Note: Compared with Sham operation group ^#^ *p* < 0.01; comparison with the MCAO group ** *p* < 0.01.

**Table 2:** Effects of each experimental group on neuron content and GSD expressions (x ± s, *n* = 6).

Group	Neuron Content (%)				GSD Expressions			
	Mean	Sd	*t* Test	Sign	Mean	Sd	*t* Test	Sign
Sham	69.67	14.08	0	^#^	0.63	0.14	0	^#^
MCAO	29	7.16	-		2.60	0.52	-	
MCAO+AS-IV	44.83	7.99	0.08		1.40	0.45	0	**
MCAO+HSYA	45.17	14.61	0.08		1.29	0.45	0	**
MCAO+AS-IV+HSYA	48	12.10	0.03	*	0.97	0.29	0	**
F	9.43				21.59			
*p*	0				0			

Note: Compared with the Sham operation group ^#^ *p* < 0.01; comparison with the MCAO group * *p* < 0.05, ** *p* < 0.01.

**Table 3:** Effects of each experimental group on MDA, SOD, IL-1β, and IL-18 levels in the ischemic cortex of rats (x ± s, *n* = 6).

Group	MDA/(mol·mg^−1^)				SOD/(mol·mg^−1^)				IL-1β (pg·mg^−1^)				IL-18 (pg·mg^−1^)			
	Mean	Sd	*t* Test	Sign	Mean	Sd	*t* Test	Sign	Mean	Sd	*t* Test	Sign	Mean	Sd	*t* Test	Sign
Sham	4.03	0.40	0	^#^	42.33	2.80	0	^#^	29.34	3.91	0	^#^	109.17	3.49	0	^#^
MCAO	8.65	0.64	-		24.17	3.19	-		24.51	3.20	-		155.23	1.60	-	
MCAO+AS-IV	7.23	0.23	0	**	32.33	4.41	0.001	**	32.60	2.48	0	**	141.74	4.87	0	**
MCAO+HSYA	6.70	0.34	0	**	31.50	3.39	0.002	**	33.99	1.94	0	**	142.34	5.75	0	**
MCAO+AS-IV +HSYA	5.93	0.16	0	**	34.50	1.38	0	**	43.54	1.59	0	**	121.15	4.53	0	**
F	110.60				25.06				43.48				110.97			
*p*	0				0				0				0			

Note: Compared with the Sham operation group ^#^ *p* < 0.01; comparison with the MCAO group ** *p* < 0.01.

**Table 4:** Effects of each experimental group on MDA, SOD, IL-1β, and IL-18 levels in the serum of rats (x ± s, *n* = 6).

Group	MDA/(mol·mg^−1^)				SOD/(mol·mg^−1^)				IL-1β (pg·mg^−1^)				IL-18 (pg·mg^−1^)			
	Mean	Sd	*t* Test	Sign	Mean	Sd	*t* Test	Sign	Mean	Sd	*t* Test	Sign	Mean	Sd	*t* Test	Sign
Sham	4.1	0.47	0	^#^	42.33	2.81	0	^#^	24.76	4.45	0	^#^	102.45	8.85	0	^#^
MCAO	5.15	0.14	-		28.83	3.37	-		32.90	2.19	-		140.33	7.92	-	
MCAO+AS-IV	5.1	0.38	1.00		32.33	3.14	0.18		29.87	0.98	0.25		123.62	2.53	0.001	**
MCAO+HSYA	4.95	0.32	0.73		32.33	2.73	0.18		34.66	2.02	0.70		140.25	5.01	1	
MCAO+AS-IV +HSYA	4.7333	0.37	0.16		33.67	3.39	0.04	*	38.50	3.77	0.01	*	138.40	6.74	0.96	
F	8.7				15.89				18.28				37.03			
*p*	0				0				0				0			

Note: Compared with the Sham operation group ^#^ *p* < 0.01; comparison with the MCAO group * *p* < 0.05, ** *p* < 0.01.

**Table 5:** Effects of each experimental group on positive expressions of NF-KB and NLRP3 in brain tissue (x ± s, *n* = 3).

Group	NF-KB				NLRP3			
	Mean	Sd	*t* Test	Sign	Mean	Sd	*t* Test	Sign
Sham	1.20	0.10	0	^#^	8.87	0.40	0	^#^
MCAO	5.23	0.25	-		27.30	1.61	-	
MCAO+AS-IV	3.67	0.67	0.002	**	21.33	2.31	0.004	**
MCAO+HSYA	3.63	0.51	0.002	**	24.00	1.00	0.009	**
MCAO+AS-IV+HSYA	2.40	0.10	0	**	16.67	2.08	0	**
F	43.56				57.41			
*p*	0				0			

Note: Compared with the Sham operation group ^#^ *p* < 0.01; comparison with the MCAO group ** *p* < 0.01.

## Data Availability

All data generated or analyzed during this study are included in this article.

## Supplementary Materials

The following supporting information can be downloaded at: https://www.mdpi.com/2076-3425/14/8/781/s1, Table S1. Effects of Each experimental group on NLRP3, GSDMD (cleaved), ASC protein expression (x ± s, n = 3). Table S2. Effects of Each experimental group on aspase-1p20 and IL-1β protein expression (x ± s, n = 3). Table S3. Effects of Each experimental group on positive expression of GSDMD and caspase-1 in brain tissue (x ± s, n = 3). Figure S1. Uncropped western blots images.
