# Supplementary material for: A Combination of Astragaloside IV and Hydroxysafflor Yellow A Attenuates Cerebral Ischemia-Reperfusion Injury via NF-κB/NLRP3/Caspase-1/GSDMD Pathway
Source: Brain Sci. 2024 Jul 31;14(8):781. doi: 10.3390/brainsci14080781 (PMC11487458; doi:10.3390/brainsci14080781)
Supplement: Supplementary file 1 [file brainsci-14-00781-s001.zip › brainsci-3036036-supplementary.pdf]

Supplementary Table S1. Effects of Each experimental group on NLRP3, GSDMD (cleaved), ASC protein expression (x±s, n=3).

Group	NLRP3/GAPDH				GSDMD (cleaved)/GAPDH				ASC/GAPDH			
	Mean	Sd	t test	sign	Mean	Sd	t test	sign	Mean	Sd	t test	sign
Sham	4.28	0.17	0	#	1.37	0.09	0	#	2.60	0.17	0	#
Model	20.47	0.34	-		5.06	0.06	-		9.94	0.02	-	
AS-IV	12.32	0.40	0	**	3.60	0.12	0	**	5.48	0.09	0	**
HSYA	8.79	0.36	0	**	2.93	0.15	0	**	7.58	0.06	0	**
AS-IV +HSYA	6.05	0.39	0	**	2.20	0.13	0	**	5.19	0.09	0	**
F	1043.83				480.15				2326.73			
P	0				0				0			

Note: Compared with Sham operation group # P < 0.01 ; Comparison with model group *P < 0.05, **P < 0.01.

Supplementary Table S2. Effects of Each experimental group on aspase-1p20 and IL-1β protein expression (x±s, n=3).

Group	Aspase-1p20 /GAPDH				IL-1β/GAPDH			
	Mean	Sd	t test	sign	Mean	Sd	t test	sign
Sham	3.17	0.11	0	#	2.83	0.12	0	#
Model	12.04	0.40	-		9.69	0.36	-	
AS-IV	7.58	0.03	0	**	5.52	0.08	0	**
HSYA	7.93	0.04	0	**	7.17	0.08	0	**
AS-IV +HSYA	5.12	0.09	0	**	3.74	0.13	0	**
F	923.85				671.02			
P	0				0			

Note: Compared with Sham operation group # P < 0.01; Comparison with model group *P < 0.05, **P < 0.01.

Supplementary Table S3. Effects of Each experimental group on positive expression of GSDMD and caspase-1 in brain tissue (x±s, n=3).

Group	GSDMD				Caspase-1			
	Mean	Sd	t test	sign	Mean	Sd	t test	sign
Sham	1.20	0.10	0	#	4.80	0.20	0	#
Model	4.33	0.15	-		8.53	0.25	-	
AS-IV	2.73	0.15	0	**	6.17	0.25	0	**
HSYA	2.87	0.32	0	**	7.30	0.30	0	**
AS-IV +HSYA	2.10	0.30	0	**	5.23	0.25	0	**
F	79.39				109.41			
P	0				0			

Note: Compared with Sham operation group # P < 0.01 ; Comparison with model group *P < 0.05, **P < 0.01.

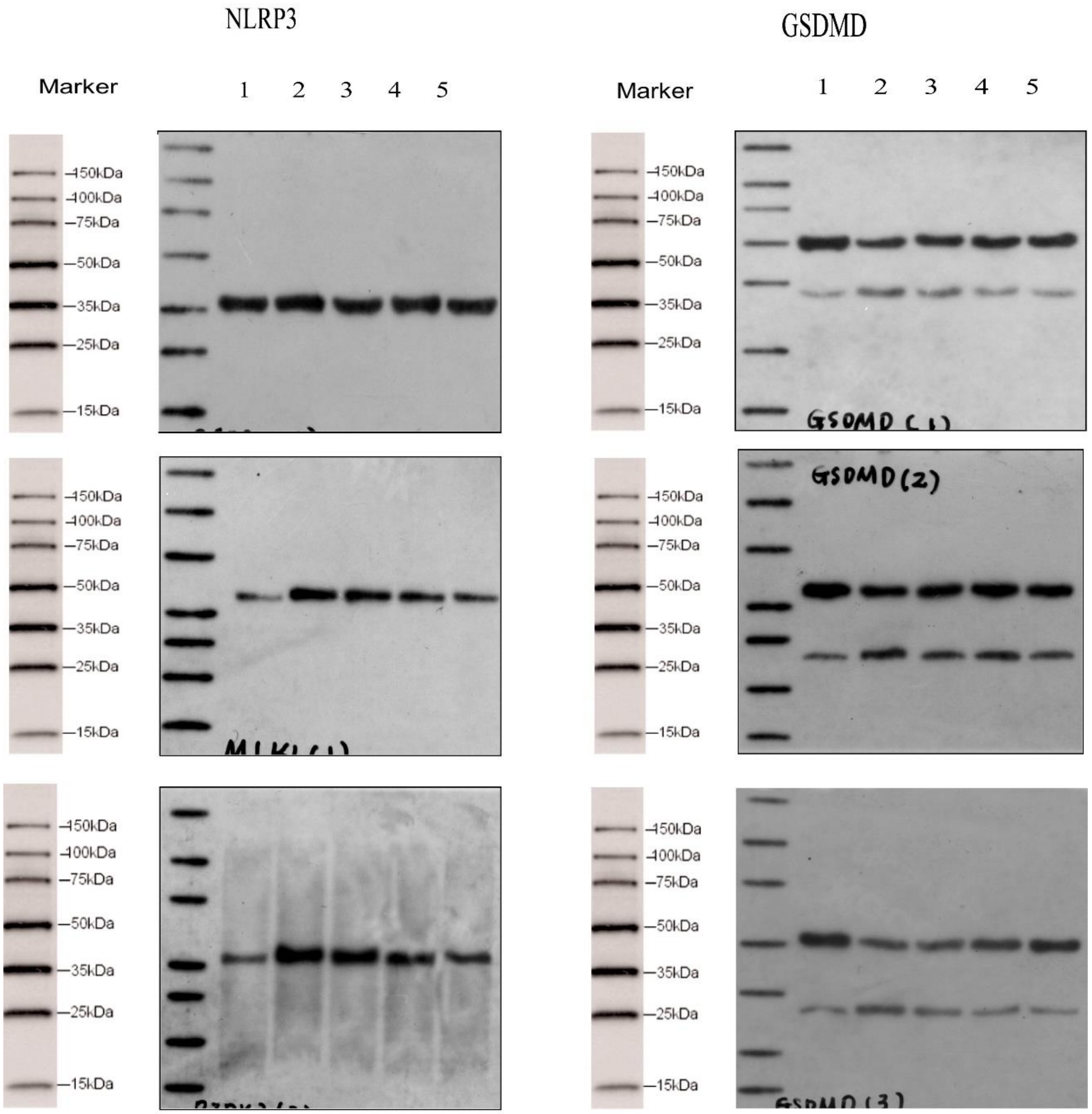

1=SHAM,2=MACO,3=AS-IV,4=HYSA,5=AS-IV+HYSA

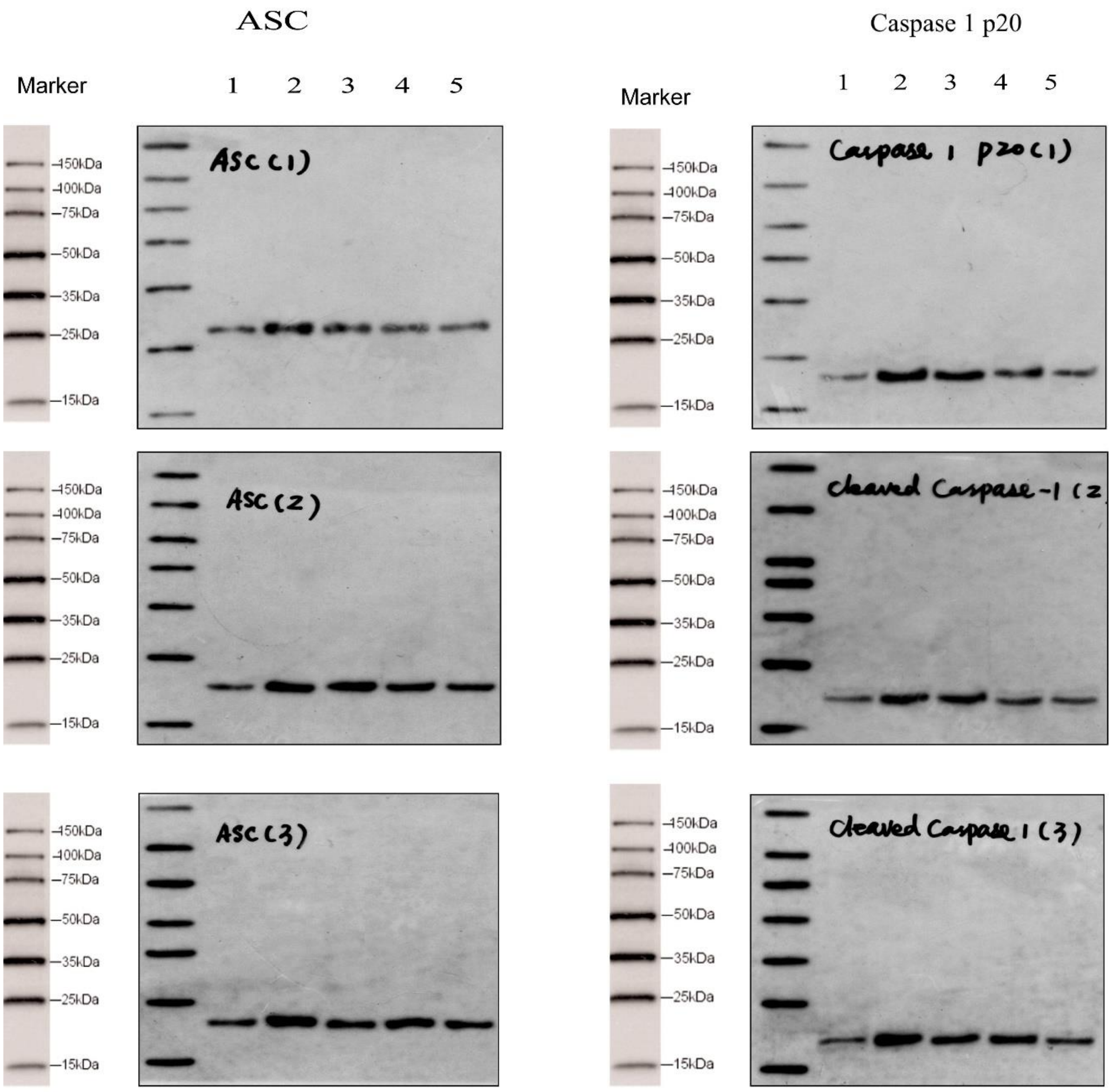

1=SHAM,2=MACO,3=AS-IV,4=HYSA,5=AS-IV+HYSA

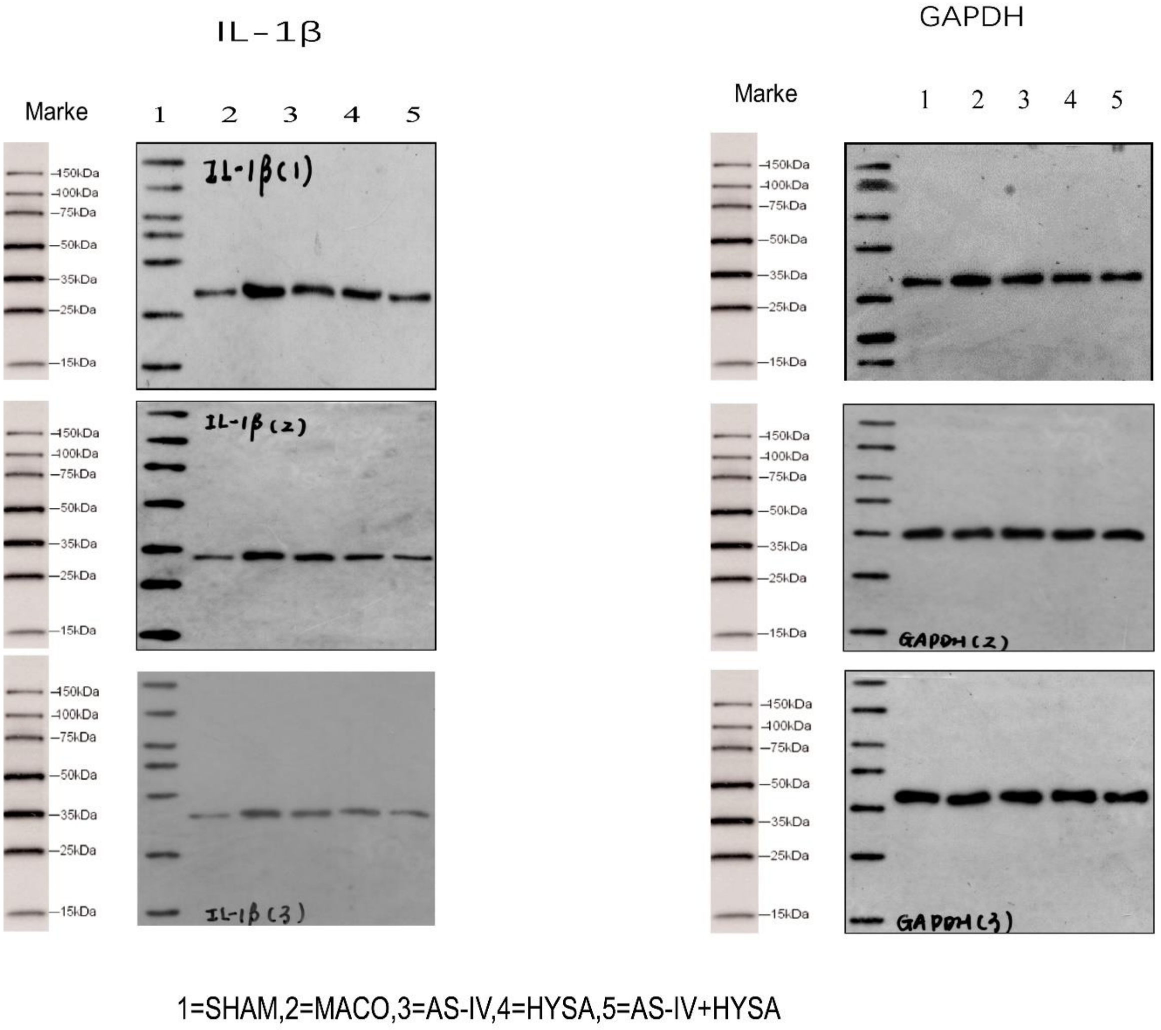

Supplementary Figure S1. Uncropped western blots images.
